# Undesirable financial effects of head and neck cancer radiotherapy during the initial treatment period

**DOI:** 10.3402/ijch.v74.26686

**Published:** 2015-01-22

**Authors:** Helen Egestad, Carsten Nieder

**Affiliations:** 1 Faculty of Health Sciences, Department of Health and Care Sciences, UiT, The Arctic University of Norway, Tromsø, Norway; 2 Department of Oncology and Palliative Medicine, Nordland Hospital, Bodø, Norway

**Keywords:** financial toxicity, head and neck cancer, radiotherapy, health economics, quality of life

## Abstract

**Background:**

Healthcare cost and reforms are at the forefront of international debates. One of the current discussion themes in oncology is whether and how patients’ life changes due to costs of cancer care. In Norway, the main part of the treatment costs is supported by general taxpayer revenues.

**Objectives:**

The objective of this study was to clarify whether head and neck cancer patients (n=67) in northern Norway experienced financial health-related quality of life (HRQOL) deterioration due to costs associated with treatment.

**Design:**

HRQOL was examined by the European Organization for Research and Treatment of Cancer (EORTC) QLQ-C30 in the beginning and in the end of radiation treatment in patients treated at the University Hospital in Northern Norway. Changes in financial HRQOL were calculated and compared by paired sample T-tests. Multiple regression analyses were used to examine correlations among gender, marital status, age and treatment with or without additional chemotherapy and changes in the HRQOL domain of financial difficulties.

**Results:**

The majority of score results at both time points were in the lower range (mean 15–25), indicating limited financial difficulties. We observed no statistically significant differences by gender, marital status and age. Increasing financial difficulties during treatment were reported by male patients and those younger than 65, that is, patients who were younger than retirement age. The largest effect was seen in singles. However, differences were not statistically significant.

**Conclusions:**

During the initial phase of the disease trajectory, no significant increase in financial difficulties was found. This is in line with the aims of the Norwegian public healthcare model. However, long-term longitudinal studies should be performed, especially with regard to the trends we observed in single, male and younger patients.

Cancer is one of the world's major diseases, a burden on patients and their families. Among others, cancer can have direct and indirect financial implications for patients and/or their families. Head and neck cancer (HNC) is the sixth most common malignancy globally, and poses a substantial economic burden to healthcare systems (1). In North America and Europe, approximately 50% of HNC patients are treated with surgery, and a combination of treatment modalities including concurrent radiotherapy and chemotherapy (2). The past decade has seen substantial changes in the treatment of HNC, with more widespread application of advancements such as robotic surgery and intensity-modulated radiotherapy (IMRT). These measures reduce treatment-related toxicity and morbidity (1). However, they may contribute to additional costs for the patients, dependent on healthcare system and insurance status. A significant amount of rehabilitation and supportive therapies are required to maintain or restore patients’ normal organ function and activities of daily living (2). Multidisciplinary rehabilitation might include nutritional support, dietary counselling, swallowing and speech therapy (1). These treatments have the ability to improve patients’ health-related quality of life (HRQOL) (3).

Previous studies have mainly focused on the costs that are driven by complex pathways and the need for involvement of several medical specialties (1). Few reports described costs associated with treatment-related side effects and follow-up care. Wissinger et al. evaluated 77 studies, mostly conducted in the USA, and found that costs are higher for HNC patients with recurrent and/or metastatic disease for patients undergoing surgery and patients insured by private payers (1). Many patients receiving cancer treatment experience both a financial burden and subjective financial distress (4). Most studies were performed in the USA, where publicly funded healthcare is limited, health insurance is linked to employment and patients may have high medical care costs (5–7). A small number of studies have focused on medical costs in countries with greater concentration of public-funded healthcare, such as the UK and Canada. These studies showed lower costs for patients (8,9). In Ireland, which has a mixed public–private healthcare system, cancer patients who were working at diagnosis experienced a drop of income, and cancer diagnoses in general caused variable amounts of out-of-pocket expenses (10). The authors of this study concluded that a complex mixed public–private healthcare system does not always provide adequate financial protection post-cancer.

The healthcare system in Norway is based on general taxpayer revenues. This means that Norwegian citizens do not pay for health insurance and have equal access to healthcare. Norwegians have to pay a small out-of-pocket amount for drugs, each medical examination or treatment. The government has set a maximum annual amount for these co-payments. The patients do not need to pay for travel cost, parking, accommodation (outpatient going through radiation treatment long way from home) or devices such as wheelchairs. The Norwegian social security system covers patients’ loss of income and their family members can apply for reimbursement of lost earnings. With this national public healthcare system, the government expects that patients should not experience related economic consequences. In other words, financial burden of cancer therapy should be absent or minimal. This study sought to examine HNC patients’ financial HRQOL during curative radiation treatment and evaluate changes regarding financial difficulties in the initial phase of treatment.

## Methods

### Study design and patient sample

This prospective study was conducted at the University Hospital in Northern Norway from May 2009 to November 2012. Sixty-seven HNC patients participated and the main results have been published earlier (11). The study was approved by the Regional Committee for Medical Research Ethics (P REK NORD 200900504-3KST017/400) and the Norwegian Social Science Data Services (21831).

### Data collection

Socio-demographic and tumour-related patient characteristics were recorded at inclusion, that is, age, gender, marital status, tumour location according to ICD-10, TNM stage (T=tumour size, N=nodal metastases, M=distant metastases) and planned treatment was registered.

Data were collected at 2 time points: at baseline which was the first week of radiation treatment, and during the last week after administration of 60 Gy. The patients filled in the European Organization for Research and Treatment of Cancer (EORTC) QLQ-C30 (12) and EORTC QLQ-H&N35 questionnaires (13). The EORTC QLQ-C30 questionnaire is a generic questionnaire for all cancers. The questionnaire is a patient-based measurement designed for self-administration which assesses multiple dimensions of HRQOL, and responses of this 30-item questionnaire are categorized into 5 functional domains (physical, role, emotional, cognitive and social) scored on a 4-point scale, one global HRQOL domain (scored on a 7-point scale), 3 symptom domains (fatigue, nausea/vomiting, pain) and 6 single items (dyspnoea, insomnia, appetite loss, constipation, diarrhoea, financial difficulties, scored on a 4-point scale). The financial question to the patients was: has your physical condition or medical treatment caused you financial difficulties? The patients could answer: not at all, a little, quite a bit or very much.

Each score was transformed into 0–100 point scale. Both EORTC instruments were scored according to recommendations in the EORTC QLQ-C30 scoring manual (14). In the 5 functional scales and the global HRQOL scale, a high score represents a high level of functioning or global HRQOL. In the symptom scales and single items, a higher score implies a high level of symptoms or problems. Regarding financial difficulties, the answer “not at all” corresponded to 0 points, “a little” to 33.33 points, “quite a bit” to 66.66 points and “very much” to 100 points. EORTC QLQ-H&N35 is a questionnaire specifically developed for HNC patients consisting of 35 items on health-related HRQOL.

### Clinical treatment

Post-operative or definitive radiotherapy was administered to the primary tumour and the regional neck lymphatics (dependent on N stage) by conventional fractionation, that is, daily dose of 2 Gy, 5 days per week. The total radiation doses were in the range of 60–70 Gy delivered over a period of 6–7 weeks. All patients were treated with three-dimensional conformal or IMRT (Table I).

**Table I:** Pretreatment baseline parameters

Patient characteristics	All n (%) N=67 (100%)
Age	
Mean (min, max)	60 (21–84)
Gender	
Male, n (%)	49 (73.1)
Female, n (%)	18 (26.9)
Marital status	
Married/partnered	33 (49.2)
Single	19 (28.4)
Missing information	15 (22.4)
Tumour location	
Oral cavity, n (%)	17 (25.4)
Pharynx, n (%)	16 (23.9)
Larynx, n (%)	16 (23.9)
Salivary glands, n (%)	7 (10.4)
Others/unknown, n (%)	11 (16.4)
T-stage (tumour size)	
T1, n (%)	21 (31.4)
T2, n (%)	20 (29.9)
T3, n (%)	8 (11.9)
T4, n (%)	8 (11.9)
Tx, n (%)	10 (14.9)
N stage (lymph nodes)	
N0, n (%)	28 (41.8)
N1, n (%)	17 (25.4)
N2, n (%)	11 (16.4)
Nx, n (%)	10 (16.4)
IMRT (intensity-modulated radiotherapy)	
Yes, n (%)	13 (19.4)
Chemotherapy	
Yes, n (%)	24 (35.8)

### Statistical analysis

In the present study, the primary outcome of interest was to examine early financial burden. Relevant information from baseline questionnaires was available in 64 patients. Changes in HRQOL were calculated and compared by paired sample T-tests. Multiple regression analyses were used to examine if baseline characteristics had any influence on changes in HRQOL. The significance level was set at p=0.05 using the statistical software SPSS 21.0 for Windows.

## Results

The baseline characteristics of the patients are presented in Table I. The mean age was 60, and 49 male and 18 female patients were included in the study. Forty-nine per cent were married and 28% were single. Dividing the tumour locations into 5 groups (oral cavity, pharynx, larynx, salivary glands and others), the most common sites of primary tumours were the oral cavity, followed by pharynx and larynx. With regard to the T-stage, 60% had T1 and T2 tumours. A minority (42%) had no lymph node metastases (N0). During the treatment period, most item scores in the EORTC-C30 declined significantly, except for emotional functional status and social functional status in female patients (Table II). In the single item questions, there were significant changes in dyspnoea, appetite and constipation during the treatment period (details not shown).

**Table II:** Changes in quality of life (EORTC-C30) from baseline to end of treatment in women and men

EORTC QLQ-C30	Baseline mean; SD	End mean; SD	Mean difference; SD	p
Physical men (n)	82.8; 17.2 (48)	68.2; 24.6 (44)	15.9; 20.2 (43)	0.000
Physical women (n)	83.3; 14.0 (16)	63.3; 25.7 (14)	20.6; 21.2 (12)	0.003
p	p = 0.913	p = 0.518	p = 0.491	
Role men (n)	73.6; 27.5 (48)	48.3; 34.6 (40)	26.5; 31.7 (39)	0.000
Role women (n)	63.5; 28.0 (16)	28.6; 30.3 (14)	43.1; 20.7 (12)	0.000
p	p = 0.211	p = 0.063	p = 0.096	
Emotional men (n)	84.3; 19.1 (48)	74.4; 24.0 (44)	10.1; 27.6 (43)	0.021
Emotional women (n)	71.9; 27.5 (16)	75.6; 23.4 (14)	−1.4; 24.1 (12)	0.538
p	p = 0.050	p = 0.873	p = 0.195	
Cognitive men (n)	86.8; 19.4 (48)	74.1; 26.5 (44)	13.4; 23.2 (43)	0.000
Cognitive women (n)	83.3; 21.1 (16)	64.3; 37.5 (14)	20.8; 31.9 (12)	0.026
p	p = 0.547	p = 0.284	p = 0.370	
Social men (n)	73.6; 27.3 (48)	62.7; 31.0 (42)	10.6; 20.3 (41)	0.002
Social women (n)	61.5; 32.6 (16)	48.8; 37.2 (14)	20.8; 46.1 (12)	0.221
p	p = 0.147	p = 0.173	p = 0.269	
Global health men (n)	67.7; 20.4 (48)	48.3; 25.8 (44)	20.3; 20.3 (43)	0.000
Global health women (n)	60.9; 30.1 (16)	45.2; 24.8 (14)	22.9; 29.5 (12)	0.012
p	p = 0.314	p = 0.001	p = 0.728	

As shown in Table III, 57 patients (89%) provided end-of-treatment data about financial difficulty. The majority of score results at both time points were in the lower range (mean 15–25), indicating limited financial difficulty. We observed no statistically significant differences by gender, marital status and age. Combined chemoradiotherapy (n=24), which is more aggressive and toxic compared to radiotherapy alone, was not associated with increasing financial difficulties. The following trends emerged: increasing financial difficulties during treatment in male patients (n=48) and those aged under 65, that is, patients who were younger than retirement age (n=43). The largest effect was seen in singles (n=16). However, the differences did not reach the level of statistical significance.

**Table III:** Changes in quality of life (EORTC-C30) from baseline to end of treatment: financial difficulty

EORTC QLQ-C30	Baseline mean; SD	End mean; SD	Mean difference; SD	p
Financial difficulty				
Men	18.1; 30.7 (48)	25.0; 32.2 (44)	−6.2; 31.9	0.210
Women	25.0; 33.3 (16)	15.4; 22.0 (13)	6.1; 20.1	0.190
p	p = 0.446	p = 0.319	p = 0.232	
Age <65 years	19.4; 30.2 (43)	24.8; 31.3 (39)	−3.7; 24.9	0.520
Age ≥65 years	20.6; 34.1 (21)	18.5; 28.5 (18)	−3.7; 39.4	0.695
p	p = 0.882	p = 0.473	p = 1.000	
Single	22.9; 33.8 (16)	33.3; 37.0 (14)	−12.8; 34.8	0.209
Married	22.2; 31.1 (33)	20.7; 27.3 (29)	−1.2; 31.3	1.000
p	p = 0.782	p = 0.213	p = 0.297	
Cisplatin and RT	20.8; 36.5 (24)	19.3; 30.1 (19)	−1.9; 38.7	1.000
RT alone	17.2; 27.0 (31)	23.0: 29.7 (29)	−4.9; 22.1	0.255
p	p = 0.674	p = 0.677	p = 0.735	

Note that not all patients provided end-of-treatment data.

## Discussion

This study mainly evaluated changes in financial HRQOL and examined if age, marital status, gender and chemotherapy modified these changes during radiation treatment in a population of HNC patients. Validated general HRQOL questionnaires were used (15,16), which also formed the basis of previous Norwegian studies in cancer patients (17,18). Bentzen et al. reported on patients previously treated for anal cancer and a comparison group of volunteers (18). Regarding financial difficulties, the mean score was 4 in volunteers and 14 in cancer survivors, p<0.001. The results for volunteers were in line with other European data, which might serve as reference values (mean scores ≤10, average 5.7) (19).

Our patients reported that most aspects of non-financial HRQOL declined significantly during the radiation treatment period, a finding which is in accordance with other studies (17,20–24). Financial difficulty did not change significantly in the treatment period. The majority of score results at both time points (start/end of radiotherapy) were in the lower range (mean 15–25), indicating limited financial difficulties. However, based on reference values even lower scores could have been expected. We observed no statistically significant differences by gender, marital status and age. However, increasing financial difficulties during treatment emerged in male patients and those younger than 65, that is, patients who were younger than retirement age. The largest effect was seen in the small subgroup of singles. However, statistical significance was not achieved when comparing subgroups. It appears understandable that singles are more vulnerable to financial problems than couples because they only have one income. In Norway, usually both partners are working, and in our study population we can expect that many patients were working at diagnosis because the mean age was 60. The social security system in Norway pays full salary from the first day the patients are unable to work and the patients and families have access to compensatory payments such as sick pay for spouse or social welfare assistance.

Financial burden of cancer therapy is a hot topic in many countries. Irrespective of healthcare system, resources are limited and threatened by increasing costs of treatment. Often, at least a proportion of costs are incurred by the patients, potentially compromising their family economy, savings and future plans. The Norwegian system aims at minimizing individual responsibility for cost of medical care and resulting consequences (25,26). Norway has the highest per capita healthcare cost of all the Nordic countries (27). These countries are similar demographically and politically, and have comparable welfare and healthcare institutions (25). The financial and economic impact of cancer is influenced by the healthcare and social welfare setting (10). We expect the same result in northern Norway as in the south of Norway because the Norwegian population has identical economic welfare. Studies from other countries illustrated the negative impact of financial burden. Wong et al. reported on a total of 400 US-American cancer patients who reviewed 2 of 3 stylized curative and non-curative scenarios that asked them to choose between 2 treatments of varying levels of efficacy, toxicity and cost (28). Each scenario included 9 choice sets. Demographics, cost concerns, numeracy and optimism were assessed. The median age of the patients was 61. Ninety-nine per cent of patients were insured. Three latent classes were identified that demonstrated (a) preference for survival, (b) aversion to high cost and (c) aversion to toxicity. Across all scenarios, patients with higher income were more likely to be in the class that favoured survival. Lower income patients were more likely to be in the class that was averse to high cost (p<0.05). Zafar et al. conducted baseline and follow-up surveys regarding the impact of healthcare costs on well-being and treatment among US-American cancer patients who contacted a national co-payment assistance foundation along with a comparison sample of patients treated at an academic medical centre (4). Among 254 participants, 75% applied for drug co-payment assistance. Forty-two per cent of participants reported a significant or catastrophic subjective financial burden; 68% cut back on leisure activities, 46% reduced spending on food and clothing and 46% used savings to defray out-of-pocket expenses. To save money, 20% took less than the prescribed amount of medication, 19% partially filled prescriptions and 24% avoided filling prescriptions altogether. In an adjusted analysis, younger age, larger household size, applying for co-payment assistance and communicating with physicians about costs were associated with greater subjective financial burden.

In contrast, Norwegian cancer patients are expected to experience much less financial consequences after diagnosis. At first sight, our results confirm this hypothesis. When interpreting our findings, the limitations of this study have to be acknowledged. The patient numbers and statistical power were limited and not all patients provided end-of-treatment data (89%). No detailed information about different aspects of personal economy was collected. Time elapsed from cancer diagnosis to end of treatment was limited (approximately 3–4 months, depending on whether surgical resection was performed before radiotherapy). Therefore, we were only able to evaluate the initial phase of the disease trajectory. Another Norwegian study assessed the impact of breast cancer on survivors’ annual income at 1–13 years of follow-up (29). The dataset contained case–control pairs, where each pair consisted of one breast cancer case and a cancer-free control, matched for age, marital status and municipality of residence. The income of breast cancer survivors had reduced immediately following diagnosis. At 1 year after diagnosis, income development between cases and controls became significantly different (p=0.006). Differences increased slightly and remained significant throughout the follow-up period. The income development of stage I breast cancer patients was similar to their controls. For higher stage breast cancer patients, the income differences were more pronounced but not always statistically significant. Ghaderi et al. analyzed long-term medical consequences of cancer at a young age (<25 years), obtained from Norwegian social security benefit records (30). Among the 5-year cancer survivors (4,031 individuals), 30% received social security benefits. The survivors had an overall 4.4 times higher risk of social security benefit uptake than the cancer-free population. The most notified causes of social security benefit uptake were diseases of the nervous system, and injury and poisoning. Taken together, several sources of information suggest that even the Norwegian health and welfare system does not guarantee absence of financial difficulties after cancer treatment. In order to rule out relevant differences in HNC patients from our region and inform healthcare authorities, larger longitudinal studies with longer follow-up are warranted.

## Conclusions

No significant financial burden was found in HNC patients who underwent radiotherapy. This is in line with the aims of the Norwegian public healthcare model. However, long-term longitudinal studies should be performed, especially with regard to the trends we observed in single, male and younger patients.
